# Uterine exteriorization versus intraperitoneal repair: effect on intraoperative nausea and vomiting during repeat cesarean delivery – A randomized clinical trial

**Published:** 2018-09

**Authors:** MS Abdellah, AM Abbas, MK Ali, A Mahmoud, SA Abdullah

**Affiliations:** Department of Obstetrics & Gynecology, Faculty of Medicine, Assiut University, Egypt. Women Health Hospital,71511,Assiut Egypt.

**Keywords:** Exteriorization, uterine incision, uterine repair, cesarean delivery, nausea, vomiting

## Abstract

**Objective:**

The current study aims to compare the rate of intraoperative nausea and vomiting after repeat cesarean delivery (CD) under two different approaches: by intraperitoneal incision repair or by uterus exteriorization for incision reapair.

**Materials and methods:**

We conducted a single-blinded randomized clinical trial (NCT03009994) at a tertiary University Hospital between the 1st of September 2016 and the 31st of December 2017. The study included pregnant women at term of gestation (>37 weeks) scheduled for repeat CD under spinal anesthesia. Women were assigned to either uterine exteriorization for incision repair (Group I) or intraperitoneal incision repair (Group II). The primary assessed was the rate of nausea and vomiting during CD.

**Results:**

The study included 1028 women in the final analysis. The rate of intraoperative nausea and vomiting was significantly lower in the intraperitoneal repair group compared to the exteriorization group (24% versus 38.7%, p= 0.001). Likewise, occurrence of uterine atony and the need for additional uterotonics were significantly lower in the intraperitoneal repair group (p= 0.001 and 0.02 respectively). Postoperatively, the rate of nausea and vomiting (12.6 % versus 21 %; P=0.001), and the time to the first recognized bowel movement (12.3 hours versus 14.1 hours; P=0.003) were significantly lower in the intraperitoneal repair group compared to the exteriorization group.

**Conclusions:**

Intraperitoneal repair of the uterine incision during repeat CD is beneficial compared to exteriorization. Improvements in the rate of intra- and postoperative nausea, vomiting, uterine atony and time to the first recognized bowel movement were observed in patients operated with this technique.

## Introduction

Cesarean delivery (CD) is the most common method of delivery in Egypt. It is applied in over 60% of all deliveries (Al Rifai, 2017). Because of this, it is imperative to practice an optimal surgical technique for CD.

Many surgical procedures aim to reduce the risk of morbidity associated with CD (Vachon-Marceau et al., 2017). During CD, uterine exteriorization, which requires to extract the uterus for a short time outside the pelvis for repairing, after placental delivery may increase infectious morbidity and postoperative pain (Xiao et al., 2014; El-Khayat et al., 2014). Due to this risk, one important challenge to improve CD is to assess the advantages of uterine incision repair techniques (CORONIS collaborative group, 2016). As such, intraperitoneal uterine repair has been reported to be more favorable with fewer complications. Therefore, intraperitoneal uterine repair must be compared to the currently applied uterine exteriorization for incision repair following CD (Siddiqui et al., 2007; Coutinho et al., 2008).

Intraoperative nausea and vomiting are common secondary complications observed during CD after uterine exteriorization. These may result from anesthetic and non-anesthetic causes (Doganay et al., 2010; Ozbay et al., 2011). Many studies in the literature already compare the existing complications between exteriorization and in-situ (intraperitoneal) uterine repair during CD. These studies assess for blood loss, intraoperative pain and return of bowel function. With this, for most studies, no significant differences exist between both techniques (Ezechi et al., 2005; Orji et al., 2008; Wahab et al., 1999). Importantly, only one study has compared the rate of nausea and vomiting occurrence after using both techniques. This study was conducted in a small patient sample (79 recruited women), thus, not encouraging the generalization of the observed results (Siddiqui et al., 2007). For this reason, a randomized controlled trial including a larger number of patients is required to investigate nausea and vomiting as primary outcomes of uterine exteriorization. This would allow to plot an explanation over the impact of choosing one technique or the other [intraperitoneal or external post CD uterine incision repair] on the risk of intraoperative nausea and vomiting (Zaphiratos et al., 2015).

The present study aims to compare the rate of nausea and vomiting associated with uterine exteriorization versus intraperitoneal uterine repair during CD as a primary endpoint.

## Materials and methods

### Study type, settings and duration

The current study is a single-blinded randomized clinical trial (NCT03009994) conducted at a tertiary University Hospital between the 1st of September 2016 and the 31st of December 2017. The Institutional Ethical Review Board approved the study. All participants signed a written informed consent before inclusion and participation in the study.

### Participants and inclusion criteria

All pregnant women scheduled for repeat CD under spinal anesthesia were invited to participate in this study. We included pregnant women with a single fetus at term of gestational age (>37 weeks) at physical status I or II, according to the American Society of Anesthesiology (ASA). The recruited women were preoperatively assessed for their for their age, parity, gestational age, and body mass index (BMI) measurement. Additionally, hemoglobin levels and hematocrit values were measured before surgery. Anemic women (Hb<8gm/dL) and those with multiple gestations, placenta praevia, premature rupture of membranes, chorioamnionitis, pre-eclampsia, diabetes mellitus, current or previous history of heart disease, liver, renal disorders or known coagulopathy and with previous repair of ruptured uterus, abdominal or pelvic surgery other than CD were excluded from the study.

### Power Calculation and Randomization

After power calculation, a sample size of 210 women was required to demonstrate a 20% decrease among the participants in the exteriorization group (P=0.05; power 80%).

Computer-generated random tables were prepared by a statistician. The groups generated were allocated in closed envelopes with a serial number. Envelopes were opened only by the obstetrician and strictly following the order of delivery of participating women just before CD. After acceptance of eligible women to participate in the study, we assigned them randomly in a 1:1 ratio to both study groups. The allocation was never changed after opening the envelopes. All patients were blinded to the allocation to avoid bias. The anesthetist was not blinded to the randomization.

### Sample size

Sample size was calculated based on the primary endpoint assessed (rate of intraoperative nausea and vomiting during CD). Based on these results, the rate of nausea and vomiting with uterine exteriorization was 29.3% (Gode et al., 2012). To be able to detect a 25% drop among the participants in the intraperitoneal repair group (P=0.05; power 80%), a sample size of 1120 women was required. (Epi-info: Centers for Disease Control and Prevention, Atlanta, GA, USA).

### Intervention

Eligible women were allocated to one of two groups. Group I (uterine exteriorization group) included women in which the obstetrician performed uterus repair by isolating the uterus out of the peritoneal cavity after delivery of the baby and placenta. Group II (intraperitoneal repair group) included women in which uterine repair was conducted via an uterine incision intraperitoneally, thus, without bringing out the uterus.

All cesarean deliveries were carried out by third-year obstetric residents trained to perform both techniques of incision repair and under the supervision the study responsible. In practice, CD was performed under spinal anesthesia, using a 25-gauge needle to inject 0.5 mg morphine and 12 mg 0.5% bupivacaine. No vasoconstrictor was used unless the blood pressure decreased by 20% from baseline values. In this case, 10 mg ephedrine was administered.

For surgery, first, a routine scrubbing of the abdominal skin with povidone-iodine was carried out followed by a Pfannenstiel incision of 10-12 cm in the skin. Then, the rectus fascia was opened, the rectus muscles separated and dissected out-off the peritoneum. The peritoneum was picked up between two tissue forceps and opened longitudinally followed by a low transverse incision to have access to the uterus. Immediately after retrieval of the fetus, perfusion of 20 IU oxytocin (Syntocinon®; Novartis Pharma, Berne, Switzerland) in 5% dextrose was initiated and all patients received 1 g of cefazolin for perioperative prophylaxis after umbilical cord clamping. Finally, the placenta was removed by the controlled cord traction method.

In both techniques, the uterine incision was closed with a continuous double layer Vicryl 0 (Ethicon; Somerville, NJ, USA) suture. The visceral peritoneum was left unsutured while the parietal peritoneum was closed with Vicryl 2-0 (Ethicon; Somerville, NJ, USA). The rectus sheath was closed with a continuous single layer of Vicryl 1 (Ethicon; Somerville, NJ, USA). Finally, the skin was closed by subcuticular Vicryl 2-0.

If uterine artery or bladder injuries ocurred during dissection and uterine incision, patients were excluded from the final analysis. Also, patients with extensive peritoneal or omental adhesions and abnormally adherent placenta (requiring additional surgical manoeuvers) were excluded from the final analysis.

### Intra-operative data collection and follow-up

During CD, intra-operative data was collected by a designated study investigator (other than the surgeon). Intra- and/or postoperative rates of nausea and vomiting were recorded for all patients. When severe nausea or vomiting was present, 10 mg of metoclopramide IV (Primperan®; Sanofi Aventis, Paris, France) was administered. Likewise, the volume of blood loss (mL) during CD was measured by adding up volumes from the suction bottle and blood-soaked sponges (minus its dry weight). Additional uterotonics or blood transfusion were also recorded.

After the procedure, postoperative data was collected in the first 24 hours. All patients were transferred to postoperative care for follow-up and received intravenous fluids (Ringer Lactate and 5% Glucose) at the rate of 100 ml/hour for 6 hours, then clear oral fluids. Abdominal auscultation with a stethoscope was done every 4 hours to assess the return of bowel movements. Resumption of bowel movements was assessed through the auscultation of bowel sounds. Intravenous infusion of Diclofenac sodium 75 mg (Voltaren®; Novartis Pharma, Berne, Switzerland) in dextrose 5% was used as an analgesic upon request. No opioid analgesia was used in our hospital. Hemoglobin and hematocrit values were measured 24 hours after CD.

Hospitalization time was calculated from the start of CD until discharge. Routine assessment of all women was carried out at 7 and 30 days after CD in our outpatient clinic. Apparition of surgical site infections and endometritis were carefully evaluated. Surgical site infection was diagnosed if purulent discharge from the incision or wound breakdown was present. Endometritis was diagnosed by sign of postoperative fever (> 38C° after the first postoperative day) with uterine tenderness, foul smelling lochia and leukocytosis (white cell count >15,000/ml).

### Study outcoumes

The primary outcome of the study was the rate of intraoperative nausea and vomiting. Nausea was defined as a subjectively unpleasant sensation associated with awareness and the urge to vomit. Vomiting was defined as the forceful expulsion of gastric contents through the mouth.

Secondary intra-operative outcomes included the mean volume of blood loss during CD, the incidence of uterine atony, the need for additional uterotonics, the need for blood transfusion, the duration of surgery, the duration of uterine incision repair and hypotension. Secondary postoperative outcomes included the mean reduction in hemoglobin and hematocrit values (difference between preoperative and postoperative levels), the need for additional analgesia, the time to first recognized bowel movements, the rate of postoperative nausea and vomiting, hospitalization time and the rate of surgical site infection and endometritis.

### Statistical analysis

Data was collected and treated with Microsoft Access database. Statistical analysis was held using the Statistical Package for Social Science (SPSS Inc., Chicago, version 22). Quantitative variables were presented as mean and standard deviation. The a paired paired-t-test analysis was used before and after CD and the Student’s t-test for comparison between both groups. Qualitative variables were presented as frequency and percentage. For these, a Chi-squared test was used for intragroup comparisons. For analysis, a p-value < 0.05 was considered significant.

## Results

### Patient randomization

**Figure 1:**
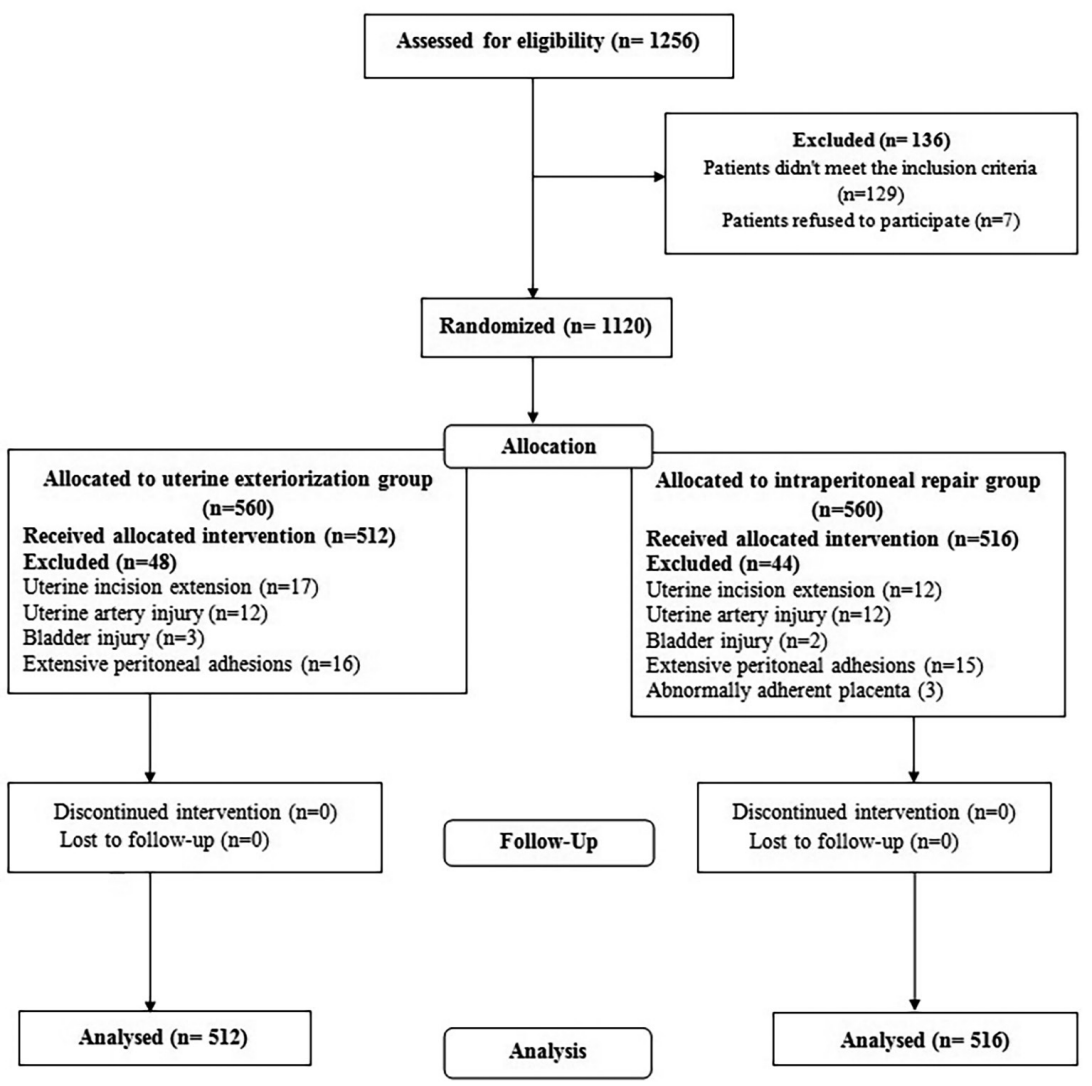
— The study flowchart.

One thousand and two hundred fifty-six women were requested to participate in the study. We excluded 129 cases as they did not meet the inclusion criteria. Seven women refused to participate in the study. The remaining 1120 women were randomized to both study groups (560 women per group). Due to intra-operative through-out surgical difficulties, 48 women in group I and 44 women in group II were excluded from the final analysis (as they did not receive the designated group intervention). Therefore, 512 women in group I and 516 women in group II were included in the final analysis (Figure 1). No significant difference was observed between the baseline characteristics after comparing both groups (Table I).

**Table I:** Baseline characteristics of the study participants.

Variables	Uterine exteriorization (n = 512)	Intraperitoneal repair (n = 516)	P-value
Age (Years)	28.34 ± 5.44	27.67 ± 5.22	0.06
Parity^$^	2 [0-4]	2 [0-5]	0.34
Maternal BMI (kg/m^2^)	27.04 ± 3.61	27.19 ± 3.38	0.69
Gestational age (weeks)	38.81 ± 0.69	38.55 ± 1.08	0.80
Type of CD ^#^			
Emergency	187 (36.5 %)	174 (33.7 %)	0.84
Elective	325 (63.5 %)	342 (66.3 %)	
N. of previous CD ^#^			
One CD	245 (47.9 %)	278 (53.9 %)	0.45
Two CD	156 (30.4 %)	143 (27.7 %)	
Three or more CD	111 (21.7 %)	95 (18.4 %)	

BMI; body mass index, CD; cesarean delivery - Data are presented as mean ± standard deviation and compared using Independent t-test.^#^ Variables are presented as frequency (percentage) and compared using Chi-square test.^$^ Variables are presented as median (minimum-maximum) and compared using Mann Whitney test.

### Surgery outcomes

The rate of intraoperative nausea and vomiting was significantly lower in the intraperitoneal repair group compared to the exteriorization group (24% versus 38.7%, p= 0.001) (Table II). Likewise, the occurrence of uterine atony and the need for additional uterotonics were significantly lower in the intraperitoneal repair group (p=0.001 and 0.02 respectively). Otherwise, no significant differences in regard to the mean blood loss volume (p=0.27), intraoperative hypotension (p=0.29) and need for blood transfusion (p=0.21) were observed between both groups. Also, the mean duration of surgery and uterine incision repair were no different between groups (p=0.36 and p=0.41, respectively).

**Table II:** The intraoperative outcomes of the study.

Variables	Uterine exteriorization (n = 512)	Intraperitoneal repair (n = 516)	P-value
Nausea and vomiting	198 (38.7 %)	124 (24 %)	0.001*
Blood loss volume (mL)	610.76 ± 165.9	576.1 ± 153.6	0.27
Uterine atony	56 (10.9 %)	17 (3.3 %)	0.001*
Need for additional uterotonics	38 (7.4 %)	11 (2.1 %)	0.02*
Hypotension	102 (19.9 %)	91 (17.6 %)	0.29
Need for blood transfusion	12 (2.3 %)	7 (1.4 %)	0.21
Duration of uterine incision repair (min)	19.73 ± 4.41	20.32 ± 4.78	0.41
Duration of CD (min)	45.16 ± 4.77	46.54 ± 6.05	0.36

Similarly, postoperative nausea and vomiting were significantly lower in the intraperitoneal repair group compared to the exteriorization group (12.6% versus 21%, p= 0.001). Plus, the need for additional analgesia was significantly higher in the exteriorization group (p=0.001). The time to the first recognized bowel movement was significantly lower in the intraperitoneal repair group compared to the exteriorization group (p=0.003). No differences between groups were observed for the mean drop of postoperative hemoglobin level (p=0.07) or hematocrit values (p=0.12).

Furthermore, time of hospitalization was similar in both groups (p=0.28). Post-surgery infection rate was lower, although no significant (p=0.62), in the intraperitoneal repair group (1.4%) compared to the exteriorization group (1.8%). Finally, the rate of postoperative endometritis was similar in both groups.

## Discussion

In the current study, we found that the rate of intra- and postoperative nausea and vomiting are significantly higher when performing exteriorization of the uterus for repairing uterine incisions following CD. Also, the rate of uterine atony, the need for additional uterotonics and analgesia as well as the time to first recognized bowel movements were also higher in the exteriorization group.

Studies have addressed the disadvantages of uterine exteriorization for repair following CD. However, the main drawbacks, usually resulting in poor evidence supporting are; first, a small sample number and second, a lack of significant outcomes (The CORONIS Trial, 2007). Therefore, we tried to overcome these obstacles by including a higher number of women in our study (1120 women) and by considering intraoperative nausea and vomiting as a primary outcome of our study.

Nausea and vomiting during CD were commonly related to fundal and peritoneal traction during exteriorization (Walsh et al., 2009). We found that intraoperative nausea and vomiting were significantly higher when the uterine repair was performed exteriorly. This observation is in line with previous studies by El-Khayat et al. (2014) and Walsh et al. (2009), however; intraoperative nausea and vomiting have been considered as a primary outcome of CD in only one study (Siddiqui et al., 2007). Others have reported that the rate of vomiting is not different between the two aforementioned surgical techniques (Gode et al., 2012; Edie-Osagie et al., 1998; Bharathi et al., 2017). Such differences may stand due to the small sample size and the nature of the recruited women behind these studies.

Uterine exteriorization was suggested, in some studies, to reduce operative blood loss and subsequently decrease the need for blood transfusion (Orji et al., 2008; Walsh et al., 2009). This may be explained by an improved visualization of the uterus during its repair but also by facilitating uterine venous drainage, thus, leading to decreased blood loss (Jacobs-Jokhan and Hofmeyr, 2004). However, our results are in accordance with Siddiqui et al. (2007) and Coutinho et al. (2008), who found that no significant differences existed between these two techniques regarding blood loss, hemoglobin and hematocrit levels (Siddiqui et al., 2007; Coutinho et al., 2008).

We also compared hypotension rates between these groups and did not find any significant increase in the rate of hypotension in the exteriorized group. Thus, in-line with Siddiaqui et al., Gode et al. and Edie-Osagie et al. who evaluated hypotension about these two surgical techniques found that the exteriorized group exhibited a non-significant increase in the incidence of hypotension. Here we demonstrate similar results.

Interestingly, we found that both groups were similar regarding the duration of uterine incision repair and the overall duration of the CD. In our hospital, we repair uterine incisions during CD without exteriorization, unlike surgeons who believe that the exteriorization of the uterus may shorten operation time. We think that exteriorization is time-consuming and may result in adnexal trauma which may be ended by catastrophic outcomes (Jacobs-Jokhan and Hofmeyr, 2004). Therefore, our results were different from Gode et al. who found a shorter surgical time in the exteriorization group (Gode et al., 2012).

Additionally, we compared the rate of uterine atony and the need of additional uterotonics between both groups. We observed a non-significant increase in the rate of uterine atony and need of additional uterotonics in the exteriorized group. To our knowledge, the exteriorization may increase the rate of uterine atony by a transient decrease in uterine blood; this event tended to disappear with the repositioning of the uterus in the abdominal cavity.

Pain reported by patients, to our mind, is also an important factor for selecting a suitable surgical technique for CD. In our study, we found a significant difference in the amount of analgesics distributed after the two surgical techniques. Similarly to Coutinho et al., we found that a small number of women requested additional analgesia in the intraperitoneal repair group.

Another significant outcome in our study was the time to the first recognized bowel movement. We observed that the time was shorter in the in the intraperitoneal group. Oriji et al. and Gode et al. also found there was a longer period for the return of bowel movement in the exteriorized group. We believe that this outcome is an essential issue affecting postoperative pain and early ambulation and hospital discharge (Abd-El-Maeboud et al., 2009).

Finally, postoperative surgical site infections and endometritis are also accounted important factors for selecting a CD surgical technique. In our study, surgical site infections and endometritis were similar in both groups. This was consistent with many studies which previously reported the same findings (Edie-Osagie et al., 1998; Orji et al., 2008; Chauhan and Devi, 2018). However, Gode et al. reported that the rate of surgical site infections was more frequent in the exteriorization group.

The strengths of our study include that it was a randomized clinical trial. Also, we were able to recruit our calculated sample size for achieving sufficient power to detect a clinically significant difference in our primary outcome. All cesarean deliveries were carried-out under a standardized procedure using same surgical steps and type of anesthesia.

However, we think that our study had some limitations such as unfeasibility to blind the surgeons regarding the procedure.

## Conclusions

The intraperitoneal technique for uterine repair during repeat CD is more advantageous than exteriorization in regards to intraoperative nausea and vomiting, the rate of uterine atony, need for additional uterotonics, postoperative nausea, and vomiting, and time to the first recognized bowel movement.
